# Social Media Use, eHealth Literacy, Disease Knowledge, and Preventive Behaviors in the COVID-19 Pandemic: Cross-Sectional Study on Chinese Netizens

**DOI:** 10.2196/19684

**Published:** 2020-10-09

**Authors:** Xiaojing Li, Qinliang Liu

**Affiliations:** 1 Center for Health and Medical Communication School of Media & Communication Shanghai Jiao Tong University Shanghai China

**Keywords:** social media, media use, COVID-19, pandemic, disease knowledge, eHealth literacy, public health, preventive behaviors

## Abstract

**Background:**

Since its outbreak in January 2020, COVID-19 has quickly spread worldwide and has become a global pandemic. Social media platforms have been recognized as important tools for health-promoting practices in public health, and the use of social media is widespread among the public. However, little is known about the effects of social media use on health promotion during a pandemic such as COVID-19.

**Objective:**

In this study, we aimed to explore the predictive role of social media use on public preventive behaviors in China during the COVID-19 pandemic and how disease knowledge and eHealth literacy moderated the relationship between social media use and preventive behaviors.

**Methods:**

A national web-based cross-sectional survey was conducted by a proportionate probability sampling among 802 Chinese internet users (“netizens”) in February 2020. Descriptive statistics, Pearson correlations, and hierarchical multiple regressions were employed to examine and explore the relationships among all the variables.

**Results:**

Almost half the 802 study participants were male (416, 51.9%), and the average age of the participants was 32.65 years. Most of the 802 participants had high education levels (624, 77.7%), had high income >¥5000 (US $736.29) (525, 65.3%), were married (496, 61.8%), and were in good health (486, 60.6%). The average time of social media use was approximately 2 to 3 hours per day (mean 2.34 hours, SD 1.11), and the most frequently used media types were public social media (mean score 4.49/5, SD 0.78) and aggregated social media (mean score 4.07/5, SD 1.07). Social media use frequency (β=.20, *P*<.001) rather than time significantly predicted preventive behaviors for COVID-19. Respondents were also equipped with high levels of disease knowledge (mean score 8.15/10, SD 1.43) and eHealth literacy (mean score 3.79/5, SD 0.59). Disease knowledge (β=.11, *P*=.001) and eHealth literacy (β=.27, *P*<.001) were also significant predictors of preventive behaviors. Furthermore, eHealth literacy (*P*=.038) and disease knowledge (*P*=.03) positively moderated the relationship between social media use frequency and preventive behaviors, while eHealth literacy (β=.07) affected this relationship positively and disease knowledge (β=–.07) affected it negatively. Different social media types differed in predicting an individual’s preventive behaviors for COVID-19. Aggregated social media (β=.22, *P*<.001) was the best predictor, followed by public social media (β=.14, *P*<.001) and professional social media (β=.11, *P*=.002). However, official social media (β=.02, *P*=.597) was an insignificant predictor.

**Conclusions:**

Social media is an effective tool to promote behaviors to prevent COVID-19 among the public. Health literacy is essential for promotion of individual health and influences the extent to which the public engages in preventive behaviors during a pandemic. Our results not only enrich the theoretical paradigm of public health management and health communication but also have practical implications in pandemic control for China and other countries.

## Introduction

### Background

COVID-19, an acute infectious disease, quickly spread worldwide after it emerged in December 2019 and has evolved from an epidemic to a pandemic. As of the end of May 2020, over 200 countries and territories had reported laboratory-confirmed cases of COVID-19, and the global number of confirmed cases of COVID-19 had exceeded 6,000,000 [1]. As a global pandemic, SARS-CoV-2, the novel coronavirus that causes COVID-19, has infected more people than either of its two predecessors, severe acute respiratory syndrome coronavirus (SARS-CoV) in 2003 and Middle East respiratory syndrome coronavirus (MERS-CoV) in 2012 [2]; thus, COVID-19 poses a serious threat to global development. There has been an obvious rise in the number of emerging and reemerging infectious diseases over the past two decades, such as severe acute respiratory syndrome (SARS, 2003), H1N1 (2009), Middle East respiratory syndrome (MERS, 2012), Ebola virus (2014), and Zika virus (2016). All these infections were difficult to control due to a lack of effective vaccines and medicines, which led to great concern and anxiety among the public and to challenges for public health systems [3,4].

Preventive behaviors are essential to control infectious diseases from both public and individual perspectives. Authorities and public health agencies should implement a variety of pharmaceutical and nonpharmaceutical interventions to prevent pandemic expansion, including vaccination and medical prophylaxis, hygienic precautions, patient isolation, and other social distancing measures [5]. Individuals should also take preventive measures to protect themselves, such as washing hands frequently with soap or hand sanitizer, avoiding crowded gatherings, and wearing face masks when going outside [6]. Because many infectious diseases erupt in a short time and have high morbidity and mortality rates, it is difficult for executive agencies to impose sufficient interventions to control these diseases in a timely fashion. Thus, effective disease-management activities benefit greatly from preventive measures by individuals [7]. Therefore, educating the public to enhance health awareness and increase disease knowledge is crucial in a pandemic.

Information communication and media use are well suited to achieve this goal by providing the public with professional information, decreasing public panic, disseminating health knowledge, and expressing appreciation to the public for their cooperation [8]. Regarding the COVID-19 pandemic, information communication is still crucial for disease prevention. China has potential advantages in the area of social media. Since the rapid development of the internet and emerging mobile media technologies, China has made remarkable achievements in mobile digital communication. Chinese internet users are also called “netizens,” defined as Chinese citizens who use the internet for at least 1 hour per week by the China Internet Network Information Center (CNNIC); these netizens have been marked by the rise of a highly connected and digitally empowered general public [9]. As of June 2019, the number of Chinese netizens had reached 847 million according to the CNNIC [10]. Social media applications are becoming increasingly diversified; WeChat, Weibo, QQ, and TikTok are the most frequently used platforms by Chinese netizens.

Also, social media is widely used by Chinese authorities to inform the public about the latest news, disseminate public health knowledge, refute rumors, and facilitate effective coordination of medical, public, and pharmaceutical resources. Although social media has been broadly used in China, the effects of social media on disease prevention have still not been greatly investigated. In this study, we hope to explore the predictive role of social media use in public preventive behaviors and how health literacy moderates the causality between individuals’ social media use and preventive behaviors during the COVID-19 pandemic in Chinese contexts.

### Literature Review and Hypotheses

The mechanisms underlying the effects of social media use on health behavioral changes is that coverage of a pandemic on social media can magnify the public’s fear and urge the public to take preventive actions [11]. Prior studies indicated that mass media use can produce positive changes or prevent negative changes in health-related behaviors across large populations [12]; for example, frequency of listening to the radio and reading the newspaper were associated with increased odds of being vaccinated [13], while time spent watching television was positively correlated with water, sanitation, and hygiene behaviors [14]. Comparatively, social media (eg, Facebook, Twitter, WeChat, Weibo) has provided the public and health institutes with new avenues for disease prevention during an epidemic or pandemic, as it allows two-way communication between health authorities and the public. Social media has also been found to be useful in terms of health-promotion interventions, such as preventing increases in risky sexual behavior [15], contributing to improved knowledge and attitudes toward skin cancer [16], positively influencing maternal influenza vaccine uptake [17], and targeting lifestyle changes among users with chronic diseases [18]. Additionally, studies on the effects of social media have shed light on its utility in public health domains. For example, Facebook was used for strategic crisis communication by health authorities in Singapore during the Zika virus pandemic [19]; moreover, WeChat and Weibo use were found to significantly increase preventive behaviors for haze health [20]. Scholars are paying increasing attention to the role of social media during pandemics; however, the question of whether social media use can affect the public’s affective responses or preventive behaviors still deserves exploration. Thus, we propose the first research question:

RQ1: Does social media use predict preventive behaviors among Chinese netizens during the COVID-19 pandemic?

Social cognitive theory is used to explain how people learn behaviors by observing others. It emphasizes the reciprocal causation of individual behaviors between personal factors (eg, values, self-efficacy, outcome expectations), behavioral factors (eg, prior behavior) and social environmental factors (eg, others’ behaviors, feedback). This theory provides a conceptual framework of how media use influences human beings’ thoughts, affect, and actions. Media use leads to behavioral changes by communicating information through two pathways. On one hand, media use promotes changes by informing, enabling, motivating, and guiding users to take direct action to effect change [21]. On the other hand, people adopt, support, spread, and share innovative ideas or behaviors in the socially mediated pathways of social media [22]. As a socially mediated factor, social media frames and reinforces social norms and enriches the ability of the public to receive health information, such as news, knowledge, and health behavior patterns. This knowledge can be rapidly and widely diffused by exerting social influences on people’s health behaviors through observational learning [23]. Therefore, the degree to which people’s use of social media to access health information for disease management may influence an individual’s health behavioral outcomes.

As media use is a composite concept that comprises a cluster of measurements, research questions about media use and health behaviors are usually presented as “how many hours did you spend on [social media platform, such as Facebook, Twitter, or YouTube] per day?”[13] or “how many times did you use a particular social media platform?” [24], which can be respectively summarized as “time of media use” (ie, how long) and “frequency of media use” (ie, how often). Time and frequency are also known to be the key variables of social media use. Thus, we proposed two hypotheses:

H1: Social media use time is positively associated with preventive behaviors during the COVID-19 pandemic.

H2: Social media use frequency is positively associated with preventive behaviors during the COVID-19 pandemic.

In addition to time and frequency, type is a crucial dimension of social media use. As the media landscape has changed dramatically, media types have rapidly become diversified in the new media environment [25]. In China, users usually obtain news or information via mobile news channels. The number of web-based news users has been reported to be 686 million, which accounts for 80.3% of Chinese netizens [10]. Web-based mobile news channels mostly consist of various applications that are characterized by social interactive functions such as reading, commenting, retweeting, and timely interaction. These platforms can be divided into different types by their functions. Official social media outlets, such as China Central Television (CCTV) and People's Daily, often serve as the voice of government or administrative institutions. Professional social media is an emerging form of social media that focuses on news in the professional domain. For example, Caixin News focuses on finance. Aggregated social media is a new type of media that collects and distributes news or information from different agencies. The scope of news on aggregated social media is widespread, including politics, the economy, culture, sports, and entertainment. Public social media (eg, WeChat, Weibo, TikTok), also called interpersonal social media, is produced and disseminated by individuals. Netizens can use public social media to share news with their friends or strangers. All the above types of social media include almost all the social media platforms in China, and each media type is aimed at particular users. For instance, traditional official media represents the official voice of the government, while public or aggregated social media provides voices to grassroots organizations or individuals [26].

At the same time, various types of social media appear to have different effects. Web-based content has been reported to facilitate safer sex literacy and information-sharing intentions on social networking sites [27]. Traditional media (eg, television and radio) can be a more effective tool for managing crises than social media and websites; meanwhile, social media should also be considered to be effective during public health interventions, as younger people heavily rely on social media to seek information [28]. Additionally, when messages are transmitted through reliable web-based personal broadcasting channels, they can induce new attitudes or intentions to change in users [29]. In particular, previous studies have examined the associations of particular types of media access with information-seeking behaviors. For example, Alhuwail and Abdulsalam [30] indicated that people searched YouTube most for health information, but they did not place a high value on other social media platforms such as Twitter, Snapchat, and Facebook. Stawarz et al [31] found in their investigation that people used mobile technologies to support their mental health for specific purposes. Hence, inspired by previous results, it is essential to examine the relationship between different social media types and the public’s preventive behaviors for COVID-19. Here, we propose another research question:

RQ2: Do social media types (official social media, professional social media, public social media, aggregated social media) differ in terms of predicting users’ preventive behaviors during the COVID-19 pandemic?

### Health Literacy and Preventive Behaviors

#### eHealth Literacy

The predictors of preventive measures are not merely based on the external impact of social media but also involve internal “assets,” including the set of health knowledge, skills, and capabilities that is called *health literacy*. As a discrete form of literacy, health literacy is becoming increasingly important in predicting health promotion and prevention [32]. In 2004, the US Institute of Medicine [33] defined health literacy as “the degree to which individuals have the capacity to obtain, process, and understand basic health information and services needed to make appropriate health decisions.” This concept is also interpreted and has evolved as a wide range of skills that people develop to seek out, comprehend, evaluate, and use health information.

The internet is now widely used and has drastically changed how health information is disseminated [34]. eHealth literacy combines information and media literacies and applies them to eHealth promotion. It has been defined as “the ability to seek, find, understand, and appraise health information from electronic sources and to apply the knowledge gained to addressing or solving health problems [35].” eHealth literacy is becoming increasingly important as individuals continue to seek medical advice from various web-based sources, especially social media. Empirical studies have also found that eHealth literacy positively influences health outcomes, such as health-promoting behaviors among people with diabetes [36] and people’s health-related quality of life [37]. College students with higher eHealth literacy were found to be less likely to consume unhealthy food [38].

#### Disease Knowledge

In addition to eHealth literacy, disease knowledge is a vital component of health literacy; it enables people to recognize the symptoms, understand the causes, and perceive the risks of chronic diseases or infectious diseases [39]. Disease knowledge is also effective in improving health management, and it even acts as a predictor of change in an individual’s health behaviors. Authorities are generally implementing additional measures to improve the level of disease knowledge among the public, with the aim of changing the attitudes of citizens toward public health prevention [40]. For example, disease knowledge can change attitudes and practices toward rabies prevention [41], levels of oncological knowledge had an impact on individuals’ decisions to consent to particular medical procedures [42], and higher public health knowledge was positively associated with more frequent handwashing [14].

Additionally, disease knowledge and eHealth literacy can combine as intermediate factors linking to health status [43]. eHealth literacy has been independently related to disease knowledge; it also further influences disease knowledge by an indirect pathway [44]. For example, diabetes knowledge was the most important factor associated with glycemic control, and health literacy through diabetes knowledge exerted an indirect influence on self-care and medication adherence [45].

Therefore, we propose four hypotheses here:

H3: eHealth literacy is positively associated with preventive behaviors during the COVID-19 pandemic.

H4: Disease knowledge is positively associated with preventive behaviors during the COVID-19 pandemic.

H5: eHealth literacy moderates the relationship between social media use and preventive behaviors during the COVID-19.

H6: Disease knowledge moderates the relationship between social media use and preventive behaviors during the COVID-19 pandemic.

**Figure 1:**
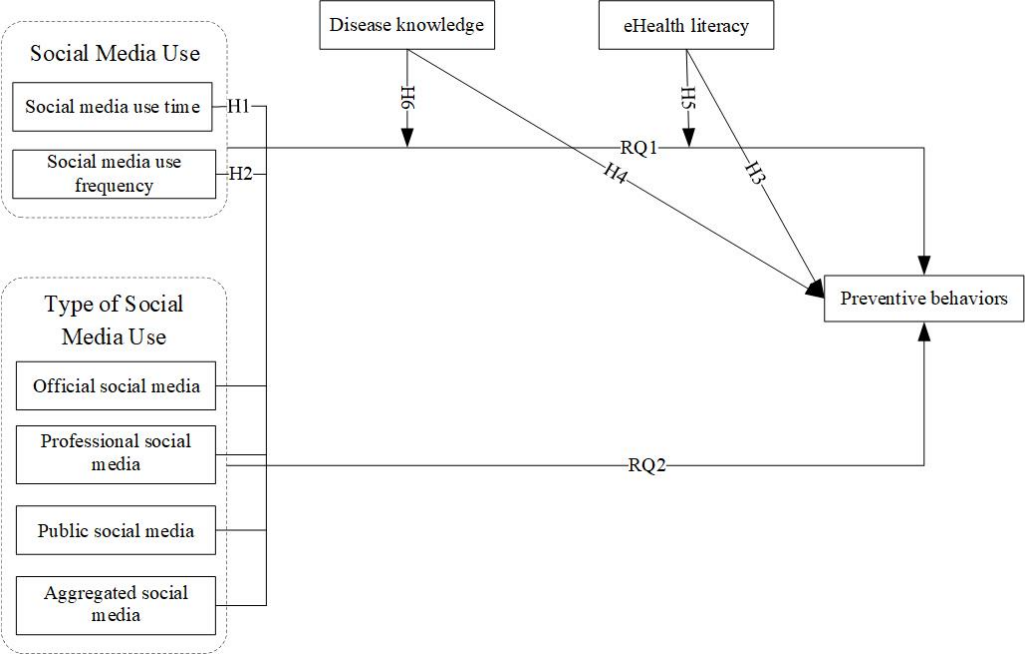
Framework map of the research questions (RQ) and hypotheses (H). SM: social media.

Figure 1 presents all the core variables and research hypotheses examined in this study.

## Methods

### Design and Recruitment

A national web-based cross-sectional survey was executed by proportionate probability sampling in this study to examine whether social media use predicted Chinese netizens’ preventive behaviors during the COVID-19 pandemic and to explore the moderators of disease knowledge and eHealth literacy. The proportionate probability sampling method was employed according to the gender and age distributions of Chinese netizens reported in the 44th Statistical Report on Internet Development of China (SRIDC) [10]. The SRIDC is an authoritative report that is released annually by the CNNIC and is based on a representative national survey with a sample size of 60,000. As the report showed, people 20 to 60 years of age were the main body of Chinese netizens; they represented 72.3% of the entire sample. In our survey, the web-based sample pool had an age limitation in that participants >60 years of age were rare. Thus, we selected 20 to 60 years of age as the target sample age range. We set the age intervals and proportions as 20 to 29 years of age (34.02%), 30 to 39 years of age (32.78%), 40 to 49 years of age (23.93%) and 50 to 59 years of age (9.27%); also, the proportions of men and women for each age range were 52.4% and 47.6%, respectively, according to the population distribution of Chinese netizens; these proportions were also in line with the SRIDC.

Participants were recruited using a web-based platform from the Questionnaire Star survey company [46], which contains over 2.6 million registered panelists in its sample pool. A structured questionnaire was developed and pretested for this study (Multimedia Appendix 1). Then, the web-based survey was partially adjusted and formally executed. The survey was conducted from February 13 to 21, 2020. After excluding ineligible samples (eg, incomplete or completed in a very short time), we finally collected 802 valid questionnaires from 952 respondents. The valid response rate was 84.24%.

### Ethics Statement

Authorization to conduct the research and recruit participants was obtained from the Institutional Review Board of the authors’ university (ID: 20200203). In addition, the purpose of this study was elucidated by the “Notification of Sample Service” (Survey ID: 57071374). Consent was obtained from all the participants before the web-based survey was conducted by the survey agency [46]. Participation was completely voluntary, and the participants could choose to quit at any time for any reason during the process of answering the web-based questionnaire.

### Instruments

#### Demographic Information

The six most frequently used sociodemographic variables were collected, including gender (0=female and 1=male), age (the respondents reported their birth year and we computed their actual age, eg, if the respondent entered “1980,” we computed 2020 – 1980 to obtain an age of 40 years), education (from 1=middle school or less to 5=master’s degree and above), monthly income (1, <¥1500; 2, ¥1500 to 3000; 3, ¥3001 to 5000; 4, ¥5001 to 8000; 5, ¥8001 to 12,000; 6, ¥12,001 to 20,000, 7, >¥20,000; 1 ¥=US $0.14), marital status (1, single; 2, divorced or widowed; 3, separated; 4, cohabiting; 5, married), and health status (from 1=severe disease to 5=good).

#### Social Media Use

Media use was measured by the following questions: social media use time (“In the past week, how much time did you spend using social media every day to learn about news of the COVID-19 pandemic?” with answers ranging from “less than one hour” to “5 hours and more”); type of social media use (“Which channel do you use often to obtain COVID-19 information every day?” with four types of social media channels, including “Official social media, such as People’s Daily,” “Professional social media, such as Ding Xiang Doctor,” “Public social media, such as WeChat,” and “Aggregated social media, such as Tencent News,” with possible answers for each social media channel of 1, never used; 2, 1 to 2 times per week; 3, 3 to 4 times per week; 4, 5 to 6 times per week, and 5, one or more times per day). Additionally, the variable of social media use frequency was measured by the sum score of the frequencies of all four types of social media channels (maximum score: 20), and a higher score indicates more frequent use of social media.

#### Preventive Behaviors

Preventive behaviors were measured by 10 items consisting of basic protective recommendations during the COVID-19 pandemic (eg, “Washing your hands after going home” and “Covering your mouth and nose with a tissue or sleeves when you cough or sneeze”). The 10 items were assessed by a self-reported measurement scale. Firstly, the measures of disease knowledge were drawn from the COVID-19 Protection Manual (Hong Kong version, February 2020) [47] and COVID-19 Protection Manual (China Mainland version, January 2020) [48]. 20 items were generated as alternative metrics. Second, we consulted with medical experts on all the metrics. According to their suggestions, we selected 10 items as the final measurement metrics. Before the formal survey was conducted, we invited 10 adults to conduct a pilot study and modified the survey correspondingly until the validity and reliability were acceptable. Finally, we adopted the adapted measures. Respondents were asked to indicate the extent to which they agreed with the statements on a 5-point Likert scale ranging from 1=never executed to 5=do it every time (Cronbach α=.75).

#### Disease Knowledge

Disease knowledge was assessed by a self-reported measurement scale consisting of 10 items (eg, “The incubation period of COVID-19 infections is generally 3-7 days, with a maximum of 14 days,” “The coronavirus volume is about 3 microns”). Like the measurement process of preventive behaviors, the instrument of disease knowledge was drawn from the COVID-19 Protection Manual (Hong Kong Version, February 2020) [47] and COVID-19 Protection Manual (China Mainland version, January 2020) [48]. We generated 20 items, also in consultation with medical experts. Finally, 10 items were used as the final measurement metrics via a pilot study. The answer options were “yes” or “no” for each item. Participants were given 1 point for the correct answer and 0 points for an incorrect response for each item. The variable of disease knowledge had possible scores of 0 to 10 (Cronbach α=.70).

#### eHealth Literacy

eHealth Literacy was assessed by the 8-item eHealth Literacy Scale (eHEALS) [34]. The eHEALS is a reliable computer-based measure of patients’ knowledge and self-efficacy for obtaining and evaluating web-based health resources. This brief scale assesses an individual’s perceived ability to find, understand, and appraise health information from web-based sources and apply that knowledge to address health concerns (eg, “I know what health resources are available on the internet” and “I know where to find helpful health resources on the internet”). The eHEALS was developed in English. It was translated into a Chinese version for our questionnaire, and we invited 5 adults to conduct a pilot study. The results indicated that the reliability of the Chinese version is high; therefore, we adopted it. Response options included a 5-point Likert scale ranging from 1=totally disagree to 5=totally agree (Cronbach *α*=.82).

### Statistical Analysis

Descriptive statistics were used to assess the sociodemographic characteristics of the respondents, including gender, age, education, monthly income, marital status, and health status. Category variables were described as n (%). Continuous variables were expressed as mean (SD). Category variables (including education, monthly income, marital status, and health status) were also dummy-coded, and one group was set as a reference group in each category. Pearson correlation analysis and hierarchical multiple regression were employed. Two-tailed Pearson correlations were used to examine the correlations between the control variables and the independent and dependent variables, respectively.

Two hierarchical regression models were used to test the research questions and hypotheses. The first hierarchical multiple regression was used to investigate RQ1, H1, H2, H3, H4, H5, and H6, in which the demographics were set as the control variables for Model 1. Then, the social media use time and social media use frequency were introduced in Model 2, and disease knowledge and eHealth literacy were introduced in Model 3. Finally, the two interaction items of social media use frequency × disease knowledge and social media use frequency × eHealth literacy were entered in Model 4. Two additional interaction items, time × eHealth literacy and time × disease knowledge, were entered in Model 5. The second hierarchical regression was carried out to explore the predictors of the four social media types (RQ2). The demographics were set as the control variables for Model 1, and four types of social media channels (official social media, professional social media, public social media, aggregated social media) were introduced in Model 2. All statistical analyses were calculated with SPSS for Windows version 22.0 (IBM Corporation).

## Results

### Descriptive Statistics

#### Sociodemographic Profiles

Among the 802 participants, 416 (51.9%) were male and 386 (48.1%) were female. The ages of the respondents ranged from 20 to 60 years, which is representative of Chinese netizens according to 2019 CNNIC statistics [10]. The sample overrepresented high education (above bachelor’s degree, 624/902, 77.7%) and high monthly income of >¥5000 (US $$736.29, 525/802, 65.3%) compared with the respective values of 9.7% and 27.1% reported in the SRIDC. Most of the respondents had a bachelor’s (undergraduate) degree or higher, and nearly half of the respondents’ monthly income was >¥8000 (US $1178). Additionally, the majority of the respondents in our sample were married (496/802, 61.8%) and in good health (486/802, 60.6%). A detailed comparison of our sample profile and the CNNIC sample is presented in Table 1.

**Table 1:** Sociodemographic characteristics of our research sample and the CNNIC sample.

Characteristic			Research sample (N=802), n (%)		CNNIC^a^ sample (N=60,000), %
**Gender**					
	Female	386 (48.1)		47.6	
	Male	416 (51.9)		52.4	
**Age (years)**					
	<20	N/A^b^		20.9	
	20-29	318 (39.7)		24.6	
	30-39	288 (35.9)		23.7	
	40-59	196 (24.4)		24.0	
	>60	N/A		6.9	
**Education**					
	Primary school and below	N/A		18.0	
	Middle school	9 (1.1)		38.1	
	High school	54 (6.7)		23.8	
	Associate degree	115 (14.4)		10.5	
	Bachelor’s degree	547 (68.1)		N/A	
	Bachelor’s degree and above^c^	N/A		9.7	
	Master’s degree and above	77 (9.6)		N/A	
**Monthly income (¥)^d^**					
	<1500	50 (6.2)		31.7	
	1500-3000	68 (8.5)		20.3	
	3001-5000	159 (19.9)		20.8	
	5001-8000	242 (30.1)		14.1	
	8001-12,000	191 (23.8)		13.0	
	12,001-20,000	78 (9.7)		N/A	
	>20,000	14 (1.7)		N/A	

^a^CNNIC: China Internet Network Information Center.

^b^N/A: not applicable.

^c^In the CNNIC survey, “Bachelor’s degree and above” was a single category.

^d^1 ¥=US $0.14 on February 13, 2020.

#### Characteristics of Social Media Use, Health Literacy, and Preventive Behaviors

Table 2 presents the basic characteristics of social media users in terms of social media use, disease knowledge, eHealth literacy, and preventive behaviors. Respondents did not spend much more time on social media every day to learn about the COVID-19 pandemic, as the average social media use time was approximately 2 to 3 hours per day (mean 2.34, SD 1.12). By contrast, the respondents used social media more often (mean score 13.59/20, SD 2.42) when compared with reference point 12. As the types of social media channels, respondents liked to use public social media and aggregated social media more than official social media and professional social media. Respondents had a high level of disease knowledge (mean score 8.15/10, SD 1.43) and eHealth literacy (mean score 3.79/5, SD 0.59). Moreover, respondents also took many preventive behaviors (mean score 4.30/5, SD 0.44) for health management during the COVID-19 pandemic.

**Table 2:** Characteristics of social media use, disease knowledge, eHealth literacy and preventive behaviors (N=802), mean (SD).

Characteristic		Value
Social media use time (hours)		2.34 (1.11)
Social media use frequency^a^		13.59 (2.42)
**Social media type^b^**		
	Official social media	2.54 (1.20)
	Professional social media	2.48 (1.11)
	Public social media	4.49 (0.78)
	Aggregated social media	4.07 (1.07)
Disease knowledge^c^		8.15 (1.43)
eHealth literacy^d^		3.79 (0.59)
Preventive behaviors^e^		4.30 (0.44)

^a^Measured by the sum score of the frequencies of all four types of social media channels (maximum score: 20).

^b^Measured on a scale with scores of 1=never used to 5=one or more times per day.

^c^Measured by 10 yes/no questions with a possible score of 1 to 10 (Cronbach α=.70).

^d^Measured by the 8-item eHealth Literacy Scale with scores of 1=totally disagree to 5=totally agree (Cronbach α=.82).

^e^Measured by a 10-item scale with scores of 1=never executed to 5=do it every time (Cronbach α=.75).

### Predictors and Moderators of Preventive Behaviors

Before the two hierarchical multiple regressions were conducted, Pearson correlations were employed to assess the correlations between independent variables and dependent variables. As displayed in the correlation table in Multimedia Appendix 2, significant correlations exist between demographics, social media use, disease knowledge, eHealth literacy, and preventive behaviors; however, social media use time (β=.07, *P*>.05) did not predict preventive behaviors. Thus, H1 was not supported.

To examine the predictors and moderators of the preventive behaviors, the first hierarchical multiple regression was carried out, and the full results are shown in Table 3 (the change in R^2^ upon adding the interaction of the last step of Model 5 was insignificant; therefore, we selected Model 4 as our final model). Social media use frequency (β=.20, *P<*.001), disease knowledge (β=.11, *P*=.001), and eHealth literacy (β=.27, *P*<.001) significantly and positively predicted preventive behaviors, respectively, when controlling sociodemographic variables (gender, age, education, monthly income, marital status, and health status). eHealth literacy (β=.27) also emerged as the main effect. These results supported H2, H3, and H4; they also partly answered RQ1, which states that social media use frequency rather than social media use time can predict preventive behaviors during the COVID-19 pandemic.

The results showed significant correlations of the social media use frequency × disease knowledge and social media use frequency × eHealth literacy interactions with preventive behaviors (β=–.07, *P*=.03, and β=.07, *P*=.04, respectively). These results indicate that disease knowledge and eHealth literacy significantly moderate the relationship between social media use frequency and preventive behaviors. Moreover, eHealth literacy positively moderated the relationship between social media use frequency and preventive behaviors, while disease knowledge negatively moderated this relationship. We also checked the moderator effects of social media use time × eHealth literacy (β=.02, *P*=.51) and social media use time × disease knowledge (β=.05, *P*=.15); however, both these correlations were insignificant. Thus, H5 and H6 were partly supported.

**Figure 2:**
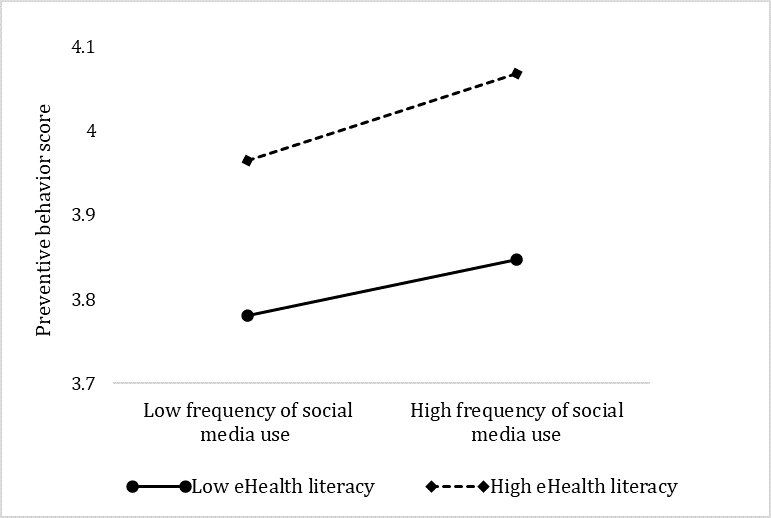
Simple slope test of the moderating effect of eHealth literacy.

**Figure 3:**
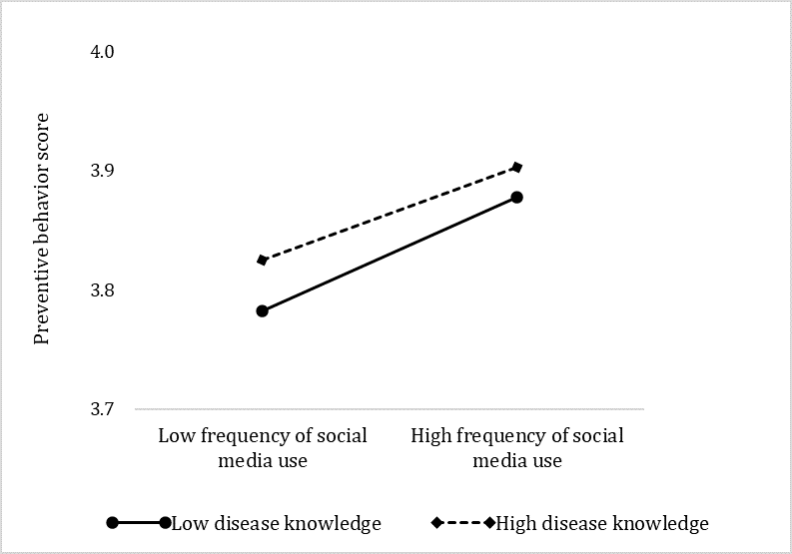
Simple slope test of the moderating effect of disease knowledge.

The slope test is often applied to test the magnitude of a moderated effect on the conditional value of a moderator. Given that the interaction items were significant, we performed slope tests and plotted the predicted preventive behaviors separately for high and low eHealth literacy or disease knowledge (1 SD above the mean and 1 SD below the mean, respectively; see Figure 2 and Figure 3). The simple slope analyses indicated that for social media users who had lower levels of eHealth literacy, a higher level of frequency of social media use (mean –1SD) was associated with higher levels of preventive behaviors (β simple=.02, *P*<.001). For people with higher levels of eHealth literacy (mean +1SD), the positive association between the frequency of social media and preventive behaviors was also significant (β simple=.044, *P*<.001), and the magnitude of this association was greater than that for lower levels of eHealth literacy.

**Table 3:** Hierarchical multiple regression examining the predictors and moderators of preventive behaviors during the COVID-19 pandemic.

Variable				Model 1^a^				Model 2^b^				Model 3^c^				Model 4^d^				Model 5^e^		
			β^f^		*P* value		β		*P* value		β		*P* value		β		*P* value		β		*P* value	
**Demographic**																						
	Female gender			–.11		.001		–.12		<.001		–.12		<.001		–.12		<.001		–.13		<.001
	Age			.20		<.001		.20		<.001		.24		<.001		.24		<.001		.23		<.001
	**Education**																					
		Middle school	Reference		N/A^g^		Reference		N/A		Reference		N/A		Reference		N/A		Reference		N/A	
		High school	.13		.13		.12		.17		.08		.33		.08		.34		.09		.29	
		Associate degree	.13		.27		.12		.29		.07		.50		.07		.54		.08		.49	
		Bachelor’s degree	.18		.25		.16		.29		.07		.63		.06		.67		.07		.62	
		Master’s degree and above	.09		.38		.08		.42		.02		.83		.01		.95		.01		.91	
	**Income (¥)^h^**																					
		<1500	Reference		N/A		Reference		N/A		Reference		N/A		Reference		N/A		Reference		N/A	
		1500-3000	.05		.34		.02		.71		.06		.24		.06		.21		.07		.17	
		3001-5000	.12		.08		.05		.44		.09		.17		.09		.16		.08		.18	
		5001-8000	.19		.01		.10		.18		.11		.11		.12		.09		.11		.10	
		8001-12,000	.23		.001		.12		.08		.13		.055		.13		.06		.13		.06	
		12,001-20,000	.17		.002		.11		.049		.10		.07		.10		.070		.10		.07	
		>20,000	.08		.06		.05		.26		.05		.23		.05		.18		.05		.17	
	**Marital status**																					
		Married	Reference		N/A		Reference		N/A		Reference		N/A		Reference		N/A		Reference		N/A	
		Single	.03		.54		.06		.21		.08		.08		.08		.07		.07		.09	
		Divorced	.01		.78		.02		.50		.03		.34		.03		.31		.03		.33	
		Separated	–.06		.09		–.05		.11		–.05		.16		–.04		.18		–.04		.18	
		Cohabiting	–.07		.05		–.05		.14		–.05		.16		–.05		.13		–.05		.12	
	**Health status**																					
		Good	Reference		N/A		Reference		N/A		Reference		N/A		Reference		N/A		Reference		N/A	
		Severe disease	–.01		.88		.01		.80		.02		.61		.01		.72		.01		.71	
		Chronic disease	–.07		.06		–.07		.03		–.07		.03		–.07		.03		–.07		.03	
		Suboptimal health	–.12		.001		–.12		<.001		–.12		<.001		–.11		.001		–.11		.001	
		Fair	–.17		<.001		–.15		<.001		–.13		<.001		–.13		<.001		–.13		<.001	
**Social media use**																						
	Time			—^i^		—		–.03		.46		–.02		.51		–.02		.46		–.03		.40
	Frequency			—		—		.25		<.001		.20		<.001		.20		<.001		.20		<.001
**Health literacy**																						
	eHealth literacy			—		—		—		—		.26		<.001		.27		<.001		.27		<.001
	Disease knowledge			—		—		—		—		.11		.001		.11		.001		.11		.001
**Interactions**																						
	1. Social media use frequency × eHealth literacy			—		—		—		—		—		—		.07		.04		.05		.11
	2. Social media use frequency × disease knowledge			—		—		—		—		—		—		–.07		.03		–.07		.03
	3. Social media use time × eHealth literacy			—		—		—		—		—		—		—		—		.02		.51
	4. Social media use time × disease knowledge			—		—		—		—		—		—		—		—		.05		.15

^a^Adjusted *R^2^*=0.11, ∆*R^2^*=0.13, *P*<.001.

^b^Adjusted *R^2^*=0.16, ∆*R^2^*=0.05, *P*<.001.

^c^Adjusted *R^2^*=0.23, ∆*R^2^*=0.07, *P*<.001.

^d^Adjusted *R^2^*=0.24, ∆*R^2^*=0.01, *P*=.01.

^e^Adjusted *R^2^*=0.24, ∆*R^2^*=0.002, *P*=.28.

^f^β: standardized regression coefficient.

^g^N/A: not applicable.

^h^1 ¥=US $0.14 on February 13, 2020.

^i^—: Not included in the model.

For disease knowledge, simple slope analyses indicate that for social media users who had lower levels of disease knowledge (mean –1SD), a higher level of social media use frequency was associated with higher levels of preventive behaviors (β simple=.060, *P*<.001). For people with higher levels of disease knowledge (mean +1SD), the positive association between frequency of social media and preventive behaviors was also significant; however, the magnitude of the association was smaller (β=.035, *P*<.001).

Concerning demographics, gender, age, monthly income, and health status were all found to significantly predict preventive behaviors. Age, monthly income, and health status positively predicted preventive behaviors. However, gender negatively predicted preventive behaviors. In detail, participants with a monthly income of more than ¥5000 engaged in more preventive behaviors than the reference group of people with a monthly income of less than ¥1500. Compared with participants who reported their health status as “good,” those who reported unhealthy status took fewer preventive measures. This suggests that social media users who were older, had higher monthly income, and had better health status were more likely to take preventive measures during the COVID-19 pandemic. Women generally engaged in more preventive behaviors than men. However, marital status and education had no significant effects on preventive behaviors.

### Types of Social Media Use and Preventive Behaviors

RQ2 focused on comparisons among the four media genres, namely official social media, professional social media, public social media, and aggregated social media. As shown in Table 4, the multiple regression results indicated that professional social media (β=.11, *P*=.002), public social media (β=.14, *P*<.001), and aggregated social media (β=.22, *P<*.001) positively predicted preventive behaviors, while official social media (β=.02, *P*=.60) did not. Furthermore, aggregated social media was found to be the highest predictor of preventive behaviors, closely followed by public social media and professional social media. However, use of official social media in China did not predict netizens’ preventive behaviors. Additionally, health literacy positively moderated the relationship between social media use and preventive behaviors.

**Table 4:** Hierarchical multiple regression examining the predicting roles of different types of social media use on preventive behaviors.

Characteristic			Model 1		Model 2	
			β^a^	*P* value	β	*P* value
**Demographic**						
	Female gender		–.11	.001	–.11	.001
	Age		.20	.000	.19	.000
	**Education**					
		Middle school	Reference	N/A^b^	Reference	N/A
		High school	.13	.130	.08	.358
		Associate degree	.13	.266	.06	.588
		Bachelor’s degree	.18	.248	.09	.560
		Master’s degree and above	.09	.384	.04	.683
	**Income (¥)^c^**					
		<1500	Reference	N/A	Reference	N/A
		1500-3000	.05	.348	.01	.909
		3001-5000	.12	.076	.05	.473
		5001-8000	.19	.011	.08	.284
		8001-12,000	.23	.001	.10	.145
		12,001-20,000	.17	.002	.09	.096
		>20,000	.08	.056	.03	.471
	**Marital status**					
		Married	Reference	N/A	Reference	N/A
		Single	.03	.542	.05	.260
		Divorced	.01	.778	.02	.606
		Separated	–.06	.086	–.04	.184
		Cohabiting	–.07	.052	–.06	.067
	**Health status**					
		Good	Reference	N/A	Reference	N/A
		Severe disease	–.01	.880	.01	.675
		Chronic disease	–.07	.056	–.06	.072
		Suboptimal	–.12	.001	–.12	.000
		Fair	–.17	.000	–.15	.000
**Social media** **type**						
	Official social media		N/A	N/A	.02	.597
	Professional social media		N/A	N/A	.11	.002
	Public social media		N/A	N/A	.14	.000
	Aggregated social media		N/A	N/A	.22	.000

^a^β: standardized regression coefficient.

^b^N/A: not applicable.

^c^1 ¥=US $0.14 on February 13, 2020.

## Discussion

This study had three goals. The first goal was to explore the predictors of preventive behaviors during the COVID-19 pandemic, the second goal was to examine the roles of disease knowledge and eHealth literacy in moderating public preventive behaviors, and the third goal was to explain the relationship between demographics and people’s preventive behaviors. The findings revealed that social media use frequency, disease knowledge, and eHealth literacy all positively predicted an individual’s preventive behaviors during the COVID-19 pandemic. Aggerated social media, public social media, and professional social media were the significant predictors of preventive behaviors within the four social media channels. Moreover, eHealth literacy positively moderated the relationship between social media use frequency and preventive behaviors, while disease knowledge negatively affected this relationship. Concerning demographics, female sex, older age, high monthly income, and good health status were likely to take more preventive measures during the COVID-19 pandemic in China.

### Social Media Use and Preventive Behaviors

For a long time, mass media (eg, television, radio, and newspapers) was recognized as an important strategy for health-promoting practice [49]. For example, a mass media campaign increased physical activity, produced positive changes, and prevented negative changes in health-related behaviors [50]. Government and executive agencies have generally used mass media and social media as convenient tools for supervising and preventing epidemics. According to the main results of this study, social media use (frequency) played a positive role in public preventive behaviors during the COVID-19 pandemic in China. This may be an important indicator of health promotion, which encourages the public to take more health measures during emergencies. Compared to mass media, social media provides the public with convenient channels to obtain news or disease knowledge and delivers information effectively. Thus, social media should be an effective strategy for public health promotion, especially during an epidemic or a pandemic.

In contrast with social media use time (which was nonsignificant), social media use frequency was a significant predictor of preventive behaviors. In other words, “how often” rather than “how long” social media was used was a good predictor of an individual’s preventive behaviors; this was an unexpected but interesting finding in this work. Time and frequency are often used to measure the regularity of social media use [51]. We attempted to draw an explanation from previous studies that investigated the relationship between social media use frequency and behavioral outcomes; we found that “frequency” may be a direct indicator of the motivations of social media use, such as self-expression, social learning, social comparison, or filtering [52,53]. Therefore, we cautiously concluded that frequency of social media use indicates the degree of engagement or investment in social media. “Frequency” may thus be a more significant predictor of social media effects.

### Types of Social Media Use and Preventive Behaviors

The positive correlation of social media use and preventive behaviors extended the study of the relationships between different types of social media use and preventive behaviors. Aggregated social media use was found to be the most significant predictor of preventive behaviors among four types of social media channels, followed by public social media and professional social media use. In contrast, official social media use was not significant. These results indicate that new media access (aggregated social media, public social media, and professional social media) deserved more attention in affecting public preventive measures than traditional media (official social media), particularly in Chinese contexts.

Aggregated social media, a novel type of news aggregator, has ensured that readers can read news stories of high quality from many outlets; this simplifies the search process of news stories and allows users to save time and effort in finding news [54]. News aggregators such as Tencent News, Sina News, and Toutiao have emerged as important components of digital content ecosystems in China, along with overseas Google News, Reddit, Bing News, etc. These aggregated social media sites have drastically changed the ways in which users access information and interact with each other. They can also generate a substitution effect when users switch from news outlets (official media) to news aggregators [55]. Consequently, aggregated social media is competing with official social media for more users’ attention and has led to an intensified propaganda crisis of official social media. This may partially explain our finding that aggregated social media was the most significant predictor for preventive behaviors among the four social media types, while official social media was not significant. Furthermore, official media outlets, such as CCTV, People's Daily and Xinhua Net, are state-driven media platforms in Chinese contexts. The content of official social media platforms mainly focuses on party ideology or party image [56]; meanwhile, the content spectrum of more extensive social imperatives is limited [57]. Therefore, the readability and humanity of public health content on official social media are lower than on aggregated social media, which may be another reason for the insignificant effect of official social media on public preventive behaviors.

Additionally, we found that public social media (eg, WeChat, Weibo, and TikTok) played a vital role in affecting users’ adoption of preventive behaviors. Because public social media is the most popular media type in China, it accelerates news diffusion among people or across regions and enables users to learn from each other [31]. On the other hand, public social media mostly disseminates information via interpersonal communication, which intensifies the perceived credibility of this type of social media [58,59]. Thus, public social media can act as a significant predictor for preventive behaviors. Finally, as an emerging web-based platform, professional medical social media sites such as Ding Xiang Doctor provide professional health knowledge with enormous medical resources and are a promising information channel for future public health emergencies.

All these results suggest that information communication during a pandemic should be built on perceived credibility or trust. Aggregated social media usually provides various sources. Users can compare different sources for a news theme and select the most trustworthy news. In contrast, media with a single source delivers only one voice and has lower perceived credibility. This media will be abandoned in a competitive context. Additionally, public social media platforms are the most popular channels of interpersonal communication in China. These platforms are usually used among acquaintances with higher levels of trust. This shows that the credibility of the information source is important for news dissemination during a pandemic. Governments should deliver more credible news and dispel rumors, which may be helpful in controlling the pandemic.

### eHealth Literacy and Disease Knowledge as Predictors and Moderators of Preventive Behaviors

Health literacy is being increasingly emphasized in public health-related studies. The relationship between health literacy and health behaviors or health status has also been highly recognized and understood based on empirical evidence. For example, it was found that poor health literacy created barriers to fully understanding individual health, illness, and treatment for people with HIV/AIDS [60]. Unimproved public mental health literacy predicted denial of self-help [61], and limited health literacy was correlated with worse health outcomes in terms of a patient’s motivation, problem-solving ability, self-efficacy, and disease knowledge, among other factors [62].

However, prior studies mainly focused on chronic disease or unhealthy lifestyles. Less attention has been paid to public health emergencies such as pandemics. In this study, we investigated if and how health literacy influenced public preventive behaviors during the COVID-19 pandemic in China. Disease knowledge and eHealth literacy were selected as the core indicators of health literacy, as concluded from previous studies [63-66]. In line with most previous findings, we verified that both disease knowledge and eHealth literacy significantly predicted Chinese respondents’ preventive behaviors during the COVID-19 pandemic. Additionally, eHealth literacy had more weight in predicting preventive behaviors than disease knowledge. Moreover, eHealth literacy positively moderated the relationship between social media use and preventive behaviors, while disease knowledge had a significant but negative effect. These findings highlight the importance of health literacy for pandemic prevention. Improving the public’s level of health literacy is essential for health promotion, not only during a pandemic but in all contexts of public health in the future.

However, it should be mentioned that health literacy is not always positively correlated with preventive behaviors. Health literacy has shown inverse effects on individuals’ healthy behaviors; for example, misinformation toward vaccination may lead to denial of the influenza vaccine [67], and a higher level of health literacy is not always associated with health-promotion behaviors [45]. This evidence underscored a compelling need to increase public awareness of health literacy in different disease conditions.

### Demographics and Preventive Behaviors

Many studies have indicated that sociodemographic indicators are vital in predicting health promotion behaviors. Our study showed similar outcomes to previous findings. We found that women engaged in more preventive behaviors than men during the COVID-19 pandemic in China. This finding may be explained by a study indicating that women are more sensitive to and interested in health information on social media than men [68]. Moreover, women usually have higher levels of disease knowledge and health literacy than men [69], and they search more frequently for health information on the internet related to changes in diet [70].

Furthermore, age, monthly income, and health status were positive predictors of preventive behaviors. These results indicate that people who are older and have higher income or good health status are more likely to take measures to prevent COVID-19, which is consistent with previous findings [67]. Additionally, education and marital status were significant predictors in the existing literature; for example, in one study [71], the odds of having accurate knowledge of malaria increased as individuals’ educational levels increased, and unmarried people were found to be more likely to have positive attitudes toward rabies prevention than married people [41]. However, these variables were not significant in this study, perhaps due to the different social contexts.

### Limitations

The results of our study should be considered in light of several limitations, and the following improvements can be implemented in future studies:

Firstly, the sample consisted of netizens between 20 and 60 years of age. Younger people (age <20 years) and older people (age >60 years) had very low response rates in the survey database. Thus, we selected 20 to 60 years of age as the target age range of our sample. People younger than 20 years or older than 60 years could be included in future studies. Furthermore, the sample consisted of much more high-income and educated netizens because our sampling was proportioned according to gender and age without consideration of income and education. Future studies are suggested to comprise netizens with lower income and less education to facilitate the generalizability of our findings.

Secondly, a single measurement of disease knowledge was used in this study, which may have led to a ceiling effect on the respondents and impaired the validity of our test. Thus, a more suitable, reasonable, and valid instrument of disease knowledge should be constructed in future studies.

Finally, this article mainly focused on the frequency and types of social media use, while other variables of media use, such as motivations and content, were not included in this study. With the rapid development of various social media platforms, such as WeChat, Weibo, Facebook, Twitter, and WhatsApp, they will continue to play a vital role in public health promotion, as we found in this study. Future research is necessary to explore how social media access affects health behaviors, including the information sources and information content accessed. Also, the experience, needs, and motivations of one’s social media use are suggested to be explored in health behavior studies in the future.

### Conclusions

Using a national web-based cross-sectional survey of a representative sample of Chinese netizens, we fully investigated our hypotheses and answered the proposed questions. We present our conclusions as follows: social media use frequency and disease knowledge and eHealth literacy were significant predictive factors of preventive behaviors; eHealth literacy and disease knowledge moderated the relationship between social media use and preventive behaviors. Aggregated social media use and public social media use were significant predictors of preventive behaviors, while official social media use was not. These results not only enrich the theoretical paradigm of public health management and health communication but also have practical implications in pandemic control both for China and for other countries.

On one hand, the confirmed predictive ability of social media use suggests that social media is helpful to disseminate pandemic news and disease knowledge, which can help the public to collectively adopt necessary preventive measures for disease control. On the other hand, the predictive ability of disease knowledge and eHealth literacy provided an endorsement that improving one’s level of health literacy is essential during a pandemic in the long term. Additionally, sociodemographic factors such as gender, age, monthly income, and health status should be taken into account in public health interventions. More attention should perhaps be paid to the people who are male, are younger, have lower income, and have poor health status during a pandemic.

## Appendix

Multimedia Appendix 1 The questionnaire used in the survey.

Multimedia Appendix 2 Pearson correlation coefficients for sociodemographic variables, social media use, disease knowledge, eHealth literacy, and preventive behaviors.

## Abbreviations

CCTV: China Central Television
CNNIC: China Internet Network Information Center
eHEALS: eHealth Literacy Scale
MERS: Middle East respiratory syndrome
MERS-CoV: Middle East respiratory syndrome coronavirus
SARS: severe acute respiratory syndrome
SARS-CoV: severe acute respiratory syndrome coronavirus
SRIDC: Statistical Report on Internet Development of China
