# Supplementary material for: Social Media Use, eHealth Literacy, Disease Knowledge, and Preventive Behaviors in the COVID-19 Pandemic: Cross-Sectional Study on Chinese Netizens
Source: J Med Internet Res. 2020 Oct 9;22(10):e19684. doi: 10.2196/19684 (PMC7581310; doi:10.2196/19684)
Supplement: Multimedia Appendix 1 [file jmir_v22i10e19684_app1.docx]

A Survey on Social Media Use and COVID-19 Prevention

Dear friends,

I’m glad to inform you that you have been selected as a participant of this random survey!

Since the outbreak of the COVID-19 pandemic, social media had played a vital role of disseminating news and disease prevention. We designed this questionnaire to explore your perceptions and use of social media in COVID-19. All the questions below are single choice or fill-in-the-blank. It will take about 10 minutes. Your truthful answers are of great value for academic work. Many thanks for your support to our study!

Health and Medical Communication Research Group

Shanghai Jiao Tong University

February 2020

**1. In the past week, how long did you use social media to learn about the news of COVID-19 pandemic every day?**

1) Less than 1 hour

2) 1-2 hours

3) 2-3 hours

4) 3-4 hours

5) 4-5 hours

6) 5 hours and above

2**. Which channel do you often use for getting the news about COVID-19 pandemic every day? Please choose the number that most applies to your situation. The bigger the number is, the more often you use it.** *(1 = never used, 5 = use one or more times a day)*

|  | never used one or more times a day | | | | |
| --- | --- | --- | --- | --- | --- |
| Social media channels | never used | *1-2* times a week | *3-4* times a week | *5-6* times a week | one or more times a day |
| 1) Official social media（like CCTV News, Peng Pai, People Daily etc.）. | 1 | 2 | 3 | 4 | 5 |
| 2) Professional social media（like Ding Xiang Doctor, Wei Yi etc.）. | 1 | 2 | 3 | 4 | 5 |
| 3) Public social media（like WeChat, Sina Weibo, TikTok etc.）. | 1 | 2 | 3 | 4 | 5 |
| 4) Aggregated social media（like Tencent News, Toutiao, NetEase News, Tencent News, Baidu news etc.）. | 1 | 2 | 3 | 4 | 5 |

3. Read the following sentences and choose the number that most fits your idea. The bigger the number is, the more you agree with it. *(1 =totally disagree, 5 =totally agree)*

|  | totally disagree totally agree | | | | |
| --- | --- | --- | --- | --- | --- |
| 1) I know what health resources are available on the Internet. | 1 | 2 | 3 | 4 | 5 |
| 2) I know where to find helpful health resources on the Internet. | 1 | 2 | 3 | 4 | 5 |
| 3) I know how to find helpful health resources on the Internet. | 1 | 2 | 3 | 4 | 5 |
| 4) I know how to use the Internet to answer my questions about health. | 1 | 2 | 3 | 4 | 5 |
| 5) I know how to use the health information I find on the Internet to help me. | 1 | 2 | 3 | 4 | 5 |
| 6) I have the skills to evaluate the health resources I find on the Internet. | 1 | 2 | 3 | 4 | 5 |
| 7) I can tell high-quality health resources from low-quality health resources on the Internet. | 1 | 2 | 3 | 4 | 5 |
| 8) I feel confident in using information from the Internet to make health decisions. | 1 | 2 | 3 | 4 | 5 |

4. Please make your judgement on the following statements, and mark *"√" or "×"* on the corresponding items.

|  | *“√”* | *“×”* |
| --- | --- | --- |
| 1）The natural host of COVID-19 is most from wild animals, such as bats, salamanders, bamboo rats, etc. | *√* |  |
| 2) There are droplets, contact, air and other "human-to-human" phenomenon in COVID-19 transmission. | *√* |  |
| 3) COVID-19 is the evolution of SARS virus. |  | *×* |
| 4) COVID-19 has a higher mortality rate than SARS. |  | *×* |
| 5) The higher degree the medical alcohol is, the better effect the sterilization has. |  | *×* |
| 6) Masks should be replaced after 4 hours. | *√* |  |
| 7) After spraying medical alcohol on the back of the mask, you could wear the mask again. |  | *×* |
| 8) Asymptom of COVID-19 doesn’t infect others. |  | *×* |
| 9) Inhalation of secondhand smoke from virus carriers may be infected with new COVID-19. | *√* |  |
| 10) COVID-19 had been confirmed "fecal-oral transmission", so we cannot eat freshwater fish later. |  | *×* |

1. How often did you take following measures for preventing COVID-19? Please choose the number that most applies to your situation. The bigger the number is, the more often you do it. *(1 = never do, 2 = occasionally do, 3 = just so so, 4 = usually do, 5 = do it every time).*

|  | never do do it every time | | | | |
| --- | --- | --- | --- | --- | --- |
| 1）Wash my hands after going home. | 1 | 2 | 3 | 4 | 5 |
| 2) Wash my hands with soap or hand sanitizer for at least 20 seconds every time. | 1 | 2 | 3 | 4 | 5 |
| 3) Wear a mask correctly when I go out. | 1 | 2 | 3 | 4 | 5 |
| 4) Cover my mouth and nose completely with a tissue or sleeves when coughing or sneezing. | 1 | 2 | 3 | 4 | 5 |
| 5) Keep the windows open for air circulation every day. | 1 | 2 | 3 | 4 | 5 |
| 6) Change my clothes as soon as I get home. | 1 | 2 | 3 | 4 | 5 |
| 7) Disinfect my mobile phone, keys, and laptop with alcohol every day. | 1 | 2 | 3 | 4 | 5 |
| 8) Thoroughly cook the meat, eggs, and various fresh food before my eating. | 1 | 2 | 3 | 4 | 5 |
| 9) Don’t go to shopping malls, supermarkets, hospitals, or other places where people usually gather recently. | 1 | 2 | 3 | 4 | 5 |
| 10) Do not contact wild animals or live birds recently. | 1 | 2 | 3 | 4 | 5 |

1. **What’s your gender？** 1）female 2）male
2. When were you born? (Fill in the blanks by yourself, such as 1980)
3. What is your final educational level?

1) Middle school or less

2) High school or secondary vocational school

3) Associate degree

4) Bachelor degree

5) Master and above

9. How about your monthly income generally?

1）Under￥1,500

2）￥1,500 - 3,000

3）￥3,001 - 5,000

4）￥5,001 – 8,000

5）￥8,001 - 12,000

6）￥12,001 – 20,000

7) ￥20,000 above

10. What is your marriage / residence status?

1) Single

2) Divorced or widowed

3) Separated

4) Cohabiting

5) Married

11. Which one is most consistent with your current health status?

1) Suffering from a severe disease (such as cancer, disability, etc.)

2) Suffering from chronic diseases (like hypertension, coronary heart disease, diabetes, arthritis, etc.)

3) Sub-health (such as fatigue, poor sleep, poor appetite, dizziness, forgetfulness, etc.)

4) Not bad (easy to get sick and the immunity is low)

5) Good (eat and sleep normally, full of energy, etc.)

**Thanks for your answers and support!**
