# Supplementary material for: Social Media Use, eHealth Literacy, Disease Knowledge, and Preventive Behaviors in the COVID-19 Pandemic: Cross-Sectional Study on Chinese Netizens
Source: J Med Internet Res. 2020 Oct 9;22(10):e19684. doi: 10.2196/19684 (PMC7581310; doi:10.2196/19684)
Supplement: Multimedia Appendix 2 [file jmir_v22i10e19684_app2.docx]

**Supplementary Table.** Pearson correlation coefficient among sociodemographic, SM use, disease knowledge, eHealth literacy, and preventive behaviors.

|  |  | *M^a^* | *SD^b^* | *1* | 2 | 3 | 4 | 5 | 6 | 7 | 8 | 9 | 10 | 11 | 12 | 13 | 14 |
| --- | --- | --- | --- | --- | --- | --- | --- | --- | --- | --- | --- | --- | --- | --- | --- | --- | --- |
| 1 | Gender | 0.52 | 0.5 | 1.00 |  |  |  |  |  |  |  |  |  |  |  |  |  |
| 2 | Age | 32.65 | 8.57 | .01 | 1.00 |  |  |  |  |  |  |  |  |  |  |  |  |
| 3 | Education=High school |  |  | .03 | .174^**^ | 1.00 |  |  |  |  |  |  |  |  |  |  |  |
| 4 | Education=Associate degree |  |  | .05 | .140^**^ | -.110** | 1.00 |  |  |  |  |  |  |  |  |  |  |
| 5 | Education=Bachelor |  |  | -.06 | -.234^**^ | -.394** | -.599** | 1.00 |  |  |  |  |  |  |  |  |  |
| 6 | Education=Master and above |  |  | .03 | .02 | -.088* | -.133** | -.477** | 1.00 |  |  |  |  |  |  |  |  |
| 7 | Income=￥1,500-3,000 |  |  | -.083* | -.172** | .03 | .00 | -.06 | .02 | 1.00 |  |  |  |  |  |  |  |
| 8 | Income=￥3,001-5,000 |  |  | -.128** | .04 | .178** | .109** | -.077* | -.141** | -.151** | 1.00 |  |  |  |  |  |  |
| 9 | Income=￥5,001-8,000 |  |  | .06 | .04 | .01 | .03 | .00 | -.04 | -.200** | -.327** | 1.00 |  |  |  |  |  |
| 10 | Income=￥8,001-12,000 |  |  | .05 | .109** | -.127** | -.095** | .137** | .03 | -.170** | -.278** | -.368** | 1.00 |  |  |  |  |
| 11 | Income=￥12,001-20,000 |  |  | .072* | .091** | -.088* | -.074* | .03 | .122** | -.100** | -.163** | -.216** | -.184** | 1.00 |  |  |  |
| 12 | Income=￥20,000 above |  |  | -.02 | .04 | -.04 | -.06 | -.113** | .280** | -.04 | -.07 | -.088* | -.075* | -.04 | 1.00 |  |  |
| 13 | Marital = Single |  |  | .01 | -.578** | -.06 | -.070* | .111** | -.04 | .249** | .00 | -.04 | -.180** | -.146** | -.092** | 1.00 |  |
| 14 | Marital = Divorced |  |  | .04 | .151** | .082* | .00 | -.02 | -.03 | .02 | -.01 | .06 | -.02 | -.03 | -.01 | -.07 | 1.00 |
| 15 | Marital = Separated |  |  | .05 | -.01 | .073* | .03 | -.04 | -.03 | .105** | -.05 | -.01 | -.06 | .05 | -.01 | -.069* | -.01 |
| 16 | Marital = Cohabiting |  |  | .04 | -.144** | .00 | .01 | .00 | .00 | -.02 | -.02 | .05 | .01 | .00 | -.03 | -.141** | -.02 |
| 17 | Health = Severe disease |  |  | .03 | .01 | .132** | -.01 | -.05 | -.01 | -.01 | -.02 | -.02 | -.02 | -.01 | -.01 | .05 | .00 |
| 18 | Health = Chronic diseases |  |  | -.01 | .194** | .073* | .04 | -.04 | -.03 | -.03 | .01 | -.03 | .04 | .02 | -.02 | -.071* | .06 |
| 19 | Health = Sub-health |  |  | -.01 | .069* | .04 | .00 | .02 | -.05 | -.04 | .02 | .01 | .03 | -.03 | .02 | -.04 | -.04 |
| 20 | Health = Not bad |  |  | -.03 | -.03 | -.03 | .01 | .01 | -.01 | -.01 | .00 | .00 | .02 | -.05 | -.04 | .02 | .02 |
| 21 | Time of SM^c^ Use | 2.34 | 1.12 | .01 | .083* | -.01 | .01 | -.04 | .073* | -.04 | -.04 | -.05 | .089* | .06 | .06 | -.114** | -.04 |
| 22 | Frequency of SM^c^ Use | 13.60 | 2.42 | .03 | .145** | -.01 | -.04 | .03 | .03 | -.136** | -.04 | .02 | .186** | .05 | .05 | -.213** | -.05 |
| 23 | Official SM^c^ | 2.54 | 1.20 | .02 | .114** | .01 | -.02 | .00 | .00 | -.082* | .01 | .01 | .120** | .00 | -.01 | -.151** | -.03 |
| 24 | Professional SM^c^ | 2.48 | 1.11 | .05 | .03 | .00 | -.082* | .03 | .07 | -.117** | .00 | -.03 | .097** | .081* | .02 | -.102** | -.077* |
| 25 | Public SM^c^ | 4.49 | 0.78 | -.07 | -.03 | -.03 | .03 | .05 | -.05 | .00 | -.03 | -.02 | .075* | -.01 | .074* | -.02 | -.06 |
| 26 | Aggregated SM^c^ | 4.07 | 1.07 | .03 | .188** | -.01 | .00 | -.01 | .03 | -.092** | -.06 | .07 | .131** | .04 | .04 | -.195** | .06 |
| 27 | Disease knowledge | 8.15 | 1.43 | -.02 | -.093** | -.06 | -.06 | .06 | .06 | -.04 | -.095** | -.02 | .07 | .05 | .03 | .03 | .02 |
| 28 | eHealth literacy | 3.79 | 0.59 | .05 | -.02 | -.06 | -.080* | .086* | .04 | -.138** | -.081* | .02 | .078* | .114** | .04 | -.088* | -.071* |
| 29 | Preventive behaviors | 4.30 | 0.44 | -.091* | .228** | .04 | -.01 | -.02 | .01 | -.089* | -.01 | .01 | .089* | .07 | .04 | -.141** | .04 |

(continuous)

|  |  |  |  | 15 | 16 | 17 | 18 | 19 | 20 | 21 | 22 | 23 | 24 | 25 | 26 | 27 | 28 | 29 |
| --- | --- | --- | --- | --- | --- | --- | --- | --- | --- | --- | --- | --- | --- | --- | --- | --- | --- | --- |
| 15 | Marital = Separated |  |  | 1.00 |  |  |  |  |  |  |  |  |  |  |  |  |  |  |
| 16 | Marital = Cohabiting |  |  | -.02 | 1.00 |  |  |  |  |  |  |  |  |  |  |  |  |  |
| 17 | Health = Severe disease |  |  | .00 | -.01 | 1.00 |  |  |  |  |  |  |  |  |  |  |  |  |
| 18 | Health = Chronic diseases |  |  | -.02 | .04 | -.01 | 1.00 |  |  |  |  |  |  |  |  |  |  |  |
| 19 | Health = Sub-health |  |  | .02 | -.01 | -.02 | -.077* | 1.00 |  |  |  |  |  |  |  |  |  |  |
| 20 | Health = Not bad |  |  | .04 | .04 | -.02 | -.085* | -.222** | 1.00 |  |  |  |  |  |  |  |  |  |
| 21 | Time of SM Use | 2.34 | 1.12 | .02 | -.078* | -.01 | -.03 | -.01 | .00 | 1.00 |  |  |  |  |  |  |  |  |
| 22 | Frequency of SM Use | 13.60 | 2.42 | -.03 | -.05 | -.082* | .04 | .05 | -.082* | .262** | 1.00 |  |  |  |  |  |  |  |
| 23 | Official SM | 2.54 | 1.20 | .01 | -.109** | -.05 | .084* | .02 | -.04 | .180** | .710** | 1.00 |  |  |  |  |  |  |
| 24 | Professional SM | 2.48 | 1.11 | .02 | .00 | -.02 | -.01 | .04 | -.06 | .171** | .597** | .308** | 1.00 |  |  |  |  |  |
| 25 | Public SM | 4.49 | 0.78 | -.079* | .02 | -.07 | -.03 | -.02 | -.01 | .104** | .344** | -.04 | -.07 | 1.00 |  |  |  |  |
| 26 | Aggregated SM | 4.07 | 1.07 | -.04 | -.01 | -.07 | .02 | .07 | -.069* | .136** | .593** | .191** | .01 | .155** | 1.00 |  |  |  |
| 27 | Disease knowledge | 8.15 | 1.43 | -.072* | .01 | .00 | -.02 | .03 | -.02 | -.02 | -.069* | -.120** | -.111** | .095** | .02 | 1.00 |  |  |
| 28 | eHealth literacy | 3.79 | 0.59 | -.01 | .00 | -.05 | .00 | -.01 | -.106** | .074* | .293** | .199** | .229** | .085* | .139** | .01 | 1.00 |  |
| 29 | Preventive behaviors | 4.30 | 0.44 | -.080* | -.110** | -.01 | .00 | -.05 | -.150** | .07 | .298** | .116** | .118** | .176** | .292** | .083* | .315** | 1.00 |

Note. Education, monthly income, marital status and health status were dummy coded, “Middle school” being the reference group for education, “Under ￥1,500” being the reference group for monthly income, “Married” being the reference group for marital status, “Good” being the reference group for health status.

^a^ M: Mean

^b^ SD: Standard Deviation

^c^ SM: social media

*p <.05, **p <.01, **p <.001
